# Effect of *Melilotus suaveolens* extract on pulmonary microvascular permeability by downregulating vascular endothelial growth factor expression in rats with sepsis

**DOI:** 10.3892/mmr.2015.3146

**Published:** 2015-01-07

**Authors:** MING-WEI LIU, MEI-XIAN SU, WEI ZHANG, YUN HUI WANG, LAN-FANG QIN, XU LIU, MAO-LI TIAN, CHUAN-YUN QIAN

**Affiliations:** 1 Department of Emergency, The First Hospital Affiliated To Kunming Medical University, Kunming, Yunnan 650032, P.R. China; 2 Surgical Intensive Care Unit, The Second Hospital Affiliated To Kunming Medical University, Kunming, Yunnan 650106, P.R. China; 3 Department of Infectious Diseases, Yan’an Hospital Affiliated To Kunming Medical University, Kunming, Yunnan 650051, P.R. China

**Keywords:** sepsis, melilotus extract tablets, vascular endothelial growth factor, microvascular permeability, rats

## Abstract

A typical indicator of sepsis is the development of progressive subcutaneous and body-cavity edema, which is caused by the breakdown of endothelial barrier function, leading to a marked increase in vascular permeability. Microvascular leakage predisposes to microvascular thrombosis, breakdown of microcirculatory flow and organ failure, which are common events preceding mortality in patients with severe sepsis. *Melilotus suaveolens (M. suaveolens)* is a Traditional Tibetan Medicine. Previous pharmacological studies have demonstrated that an ethanolic extract of *M. suaveolens* has powerful anti-inflammatory activity and leads to an improvement in capillary permeability. However, the mechanisms underlying its pharmacological activity remain elusive. The present study aimed to assess the impact of *M. suaveolens* extract tablets on pulmonary vascular permeability, and their effect on regulating lung inflammation and the expression of vascular endothelial growth factor (VEGF) in the lung tissue of rats with sepsis. A cecal ligation and puncture (CLP) sepsis model was established for both the control and treatment groups. ~2 h prior to surgery, 25 mg/kg of *M. suaveolens* extract tablet was administered to the treatment group. Polymerase chain reaction and western blot analyses were used to assess the expression of nuclear factor (NF)-κB and VEGF in the lung tissue, and ELISA was applied to detect changes in serum tumor necrosis factor-α as well as interleukins (IL) -1, -4, -6, and -10. The lung permeability, wet/dry weight ratio and lung pathology were determined. The results demonstrated that in the lung tissue of CLP-rats with sepsis, *M. suaveolens* extract inhibited the expression of NF-κB, reduced the inflammatory response and blocked the expression of VEGF, and thus significantly decreased lung microvascular permeability. The effects of *M. Suaveolens* extract may be of potential use in the treatment of CLP-mediated lung microvascular permeability.

## Introduction

Sepsis, a systemic inflammatory response syndrome caused by infection, is known to be accompanied by the presence of bacteria, which may arise from a highly virulent focus of infection. Sepsis may often cause acute lung injury (ALI) (1,2). When ALI occurs, cytokines, chemokines, adhesion molecules and other inflammatory mediators are produced in the endothelial cells activated within the pulmonary vasculature, which destroys the integrity of pulmonary vascular endothelial cells. This leads to increased permeability of capillaries in the lung, and consequent pulmonary edema (1), which results in acute respiratory distress syndrome, multiple organ failure, high mortality and other major problems in intensive care. The reduction of pulmonary capillary permeability is therefore of marked clinical importance. Vascular endothelial growth factor (VEGF) is one of the most important regulatory factors during vascular formation. Upon activation of inflammation, alveolar macrophages and neutrophils are able to release a large amount of VEGF (3,4), which most commonly increases the permeability of post-capillary venules. *In vitro* studies have demonstrated that the effect of VEGF on increasing vascular permeability is ~20,000 times more potent than that of histamine (5,6). In the early stage of ALI, neutrophils, monocytes, macrophages and platelets activated by inflammation synthesize and release large amounts of VEGF, leading to high vascular permeability and consequently to pulmonary edema (6). *Melilotus suaveolens* Ledeb *(M. suaveolens)*, a Traditional Tibetan Medicine also known as *Melilotus suavcolen* or wild alfalfa, is a bitter, ‘cold-tasting’ herb which has proven to be effective in fever reduction, detoxification, anti-inflammatory and drying limbs ichor (7). It is therefore traditionally applied to treat a range of illnesses, including spleen disease, twisted intestinal fever, diphtheria and tonsillitis (8). Currently, there are few published studies on the effects of *M. suaveolens*. Pharmacological studies have demonstrated that an ethanolic extract from *M. suaveolens* has a powerful anti-inflammatory activity (9), which inhibited formaldehyde and propylene glycol-induced capillary permeability, and was effective in the improvement of blood circulation (10). Therefore, downregulating the expression of VEGF in the lung may be a mechanism by which *M. suaveolens* reduces pulmonary capillary permeability. The aim of the present study was to investigate the mechanism of action by which *M. suaveolens* affects CLP-induced pulmonary capillary permeability in rats, and to establish whether this effect occurs by regulating VEGF expression.

## Materials and methods

### Mice

Male Sprague-Dawley mice were purchased from Kunming Medical University Laboratory Animal Center (Kunming, China). All of the mice were housed in the Kunming Medical University Animal Care Facility and were maintained under pathogen-free conditions. The mice were 8–9 weeks of age at the initiation of the experiment and were maintained on a standard laboratory diet and water *ad libitum*. The experimental procedures were approved by the Committee of Animal Experimentation of the Kunming Medical University (Kunming, China).

### Reagents

A reverse transcription reaction kit was purchased from Takara Biotechnology Co. Ltd. (Dalian, China); TRIzol was from Invitrogen Life Technologies (Carlsbad, CA, USA) and electrophoresis reagents were from Promag Co. (Ningbo, China); an RT Reaction kit was obtained from Takara Biotechnology Co. Ltd (Dailan, China); a PCR Amplification Reagent kit and the 100 bp DNA ladder marker were obtained from Sangon Biological Engineering Co. Ltd. (Shanghai, China); GAPDH was obtained from Santa Cruz Biotechnology, Inc. (Santa Cruz Biotechnology, Inc., Santa Cruz, CA, USA); rabbit anti-mouse NF-κB and VEGF polyclonal antibodies were purchased from Wuhan Boster Biological Technology, Ltd. (Wuhan, China); *M. suaveolens* Extract Tablets were from Seiko Eiyo Yakuhin Co. Ltd. (Osaka, Japan); fluorescein isothiocyanate (FITC)-albumin and hexadecyl-trimethyl-ammonium bromide were purchased from Sigma-Aldrich (St. Louis, MO, USA) and SYBR green I was obtained from Biotium (Hayward, CA, USA). The Oligo (dT18) and primers were synthesized by Shanghai Invitrogen (Shanghai, China). The dNTP was obtained from Promega Corp. (Madison, WI, USA).

### Animal model of sepsis

All of the studies were performed on rats with an average weight of 40.4 g. To induce sepsis, the rats were anesthetized with isoflurane (4% induction, 2% maintenance) and placed on a warming pad. Following laparotomy, the cecum was exteriorized, and the membrane between the cecum and the mesentery was carefully cut to release the cecum. The cecum was ligated 1.5 cm from the tip or just below the ileocecal valve with 4-0 silk. Two punctures were made with an 18-gauge needle and 1 mm of fecal material was expressed from the punctures. The incision was sutured in two layers with 4-0 silk. In the sham animals, the cecum was located but neither ligated nor punctured. The animals were resuscitated with 3 ml/100 g body weight normal saline subcutaneously immediately following surgery.

### Grouping and treatment

According to a random number table, 88 rats were randomly divided into four groups: Normal control group, sham operation group (sham group), sepsis model group [(untreated) sepsis group] and *M. suaveolens* treatment group (treatment group), with 22 rats in each group. The model group and treatment group were induced by cecal ligation and puncture (CLP) and, 2 h prior to surgery, were administered the *M. suaveolens* extract via tube, at a dose of 25 mg/kg every 8 h. The normal control group, sham group and (untreated) sepsis group were subject to treatment with the same volume of normal saline. A total of 22 rats in each group were anesthetized using ether at each of the following time-points 24 h post-surgery. Subsequently, the right internal carotid artery was isolated. Blood was collected via the orbital sinus. EDTA was used as an anti-coagulant, and the plasma was isolated by centrifugation at 10,000 × g for 5 min. All the animals were sacrificed 24 h following surgery via anesthesia with ether and lung tissues were collected, washed with saline solution, dried with filter paper and weighed. The plasma and tissues were stored at −20°C for subsequent experiments.

### RNA isolation and quantitative polymerase chain reaction (qPCR)

The left lung tissues were homogenized in TRIzol reagent using a Mixer Mill 301 (Tianjin Tian Chang Technology Co., Ltd., Tianjin, China). The total RNA was extracted using TRIzol reagent according to the manufacturer’s instructions and quantified spectrophotometrically. A total of 2 μg RNA from each sample was added to a total volume of 25 μl reaction mixture containing 2.5 μM oligo (dT) primer (Promega Corp.; cat. no. C110A), and 200 U Molony murine leukemia virus reverse transcriptase (M-MLV; Promega Corporation; cat. no. M5314). The reaction was initiated by incubating the reaction mixture for 1 h at 42°C for reverse transcription and stopped by heating for 10 min at 70°C. An aliquot (0.5 μl) of each reverse transcription product was added to 20 μl reaction mixture containing LightCycler-FastStart DNA Master SYBR Green I, 0.5 μM of each primer corresponding to mouse NF-κB and VEGF or GAPDH, and 4 mM MgCl_2_ to amplify the genes in a LightCycler (Roche, Mannheim, Germany). For reverse transcription PCR, 1 μg of total RNA from each sample was resuspended in 25 μl final volume of reaction buffer. GAPDH was used as an internal control. The following primers were used for PCR: VEGF forward primer, 5′-GCTCTCTTGGGTGCACTGGA-3′ and reverse primer, 5′-CACGCCTTGGCTTGTCACCA-3′; NF-κB forward primer, 5′-GCACGGATGACAGAGGCGTGTATAAGG-3′ and reverse primer, 5′-GGCGGATGATCTCCTTCTCTCTGTCTG-3′; and GAPDH forward primer, 5′-AAT GCA TCC TGC ACC ACC AA-3′ and reverse primer, 5′-GTA GCC ATA TTC ATT GTC ATA-3′. Following pre-incubation at 95°C for 10 min, the PCR was performed as follows: 35 cycles of denaturation at 95°C for 15 sec, annealing at 60°C for 5 sec and elongation at 72°C for 12 sec. The 2^−[ΔΔCt]^ method was used to compare the mRNA expression levels of genes in the experimental groups with the control groups.

### Western blot analysis

The lung tissues were snap-frozen in liquid nitrogen, pulverized and resuspended in ice-cold lysis buffer (Solarbio Science & Technology, Beijing, China). Protein concentrations were determined with the Bradford method. Lysates were allowed to solubilize on ice for 30 min and particulate mass was removed by centrifugation (15,000 × g) for 15 min at 4°C. The supernatants were analyzed by SDS-PAGE. Primary antibodies used included rabbit anti-VEGF monoclonal antibody (1:400), rabbit anti-NF-κB65 monoclonal antibody (1:400) (Boster Biological Technology, Ltd) and mouse anti- GAPDH monoclonal antibody (1:400) (Santa Cruz Biotechnology, Inc). The secondary antibodies were horseradish peroxidase-labeled antibodies (Pierce; Thermo Scientific, Rockford, IL, USA). The blots were processed for enhanced chemifluorescence using a Pierce ECL Western blotting substrate (Pierce; Thermo Scientific).

### Immunohistochemistry

Immunostaining was performed on the lung sections following antigen retrieval using Retrievagen A (Zymed Laboratories, Inc., San Francisco, CA, USA) at 100°C for 20 min, and quenching endogenous peroxidases with 3% H_2_O_2_. The sections were blocked with 2% bovine serum albumin (BSA) in phosphate-buffered saline (PBS) followed by staining with primary anti-VEGF-α and anti-NF-κBp65 (BD Pharmingen, San Jose, CA, USA) at room temperature for 1 h. The sections were washed and following application of secondary antibody (R&D Systems, Minneapolis, MN, USA), tissues were developed using Vectastain ABC (Vector Laboratories, Inc., Burlingame, CA, USA) and 3,3′-diaminobenzidine (Vector Laboratories, Inc.). Following staining, five high-power fields (x200) were randomly selected in each slide, and the average proportion of positive expression in each field was counted using the true color multi-functional cell image analysis management system (Image-Pro Plus; Media Cybernetics, Rockville, MD, USA), and expressed as positive unit (PU).

### Cytokine and VEGF measurements in bronchoalveolar lavage (BAL) and plasma

Mice were sacrificed after 24 h of treatment, and BAL was performed via the tracheal catheter in the right lung lobes using 0.8 ml PBS; the withdrawn fluid was centrifuged, and the supernatant was snap frozen and stored at −80°C for further use. Aliquots of BAL fluid and plasma were detected in duplicate by ELISA (ELISA kit offered by Glory Science Co., Ltd., Del Rio, TX, USA) kits for tumor necrosis factor-α (TNF-α), interleukin (IL)-1β, IL-6 and IL-10 according to the manufacturer’s instructions.

### Pulmonary vascular permeability assays

Two hours prior to sacrification, FITC-labeled albumin (5 mg/kg body weight) was administered via tail-vein injection at 6 and 24 h. Immediately following sacrification, the lungs were lavaged three times with PBS (0.5 ml per lavage) and the samples combined. Fluid recovery was ~95%. The BAL samples were centrifuged at 3,000 × g for 10 min. FITC fluorescence in the BAL fluid was measured using a 960CRT computer controlled fluorescence spectrophotometer (Shanghai Tiancheng Technology Co., Ltd., Shanghai, China) with excitation at 484 nm and emission at 510 nm.

### Wet/dry (W/D) lung weight ratio and water content

W/D weight ratio was used as an index of tissue water content. 24 h following administration of *M. suaveolens* extract, the animals were anesthetized using ketamine (80 mg/Kg i.p.) and xylazine (20 mg/Kg i.p.), sacrificed and lungs were excised en bloc. The lung lobes were cut, blot dried and placed on pre-weighed glass plates. The wet weight of the tissue was determined immediately. The tray with the tissue was then baked in an oven at 55°C for 72 h to obtain a constant weight. After the dry weight of the tissue was determined, the wet/dry (W/D) lung weight ratio was calculated. The lung water content was calculated as the wet weight minus dry weight and wet weight ratio of lung tissue multiplied by 100%.

### Pathological observation of lung tissues

The middle lobe of the right lung was fixed by infusing 10% formaldehyde solution in the same pressure, and the inflation of the lung was kept uniform. The tissue was then embedded in paraffin wax, cut into sections and stained with hematoxylin-eosin (H&E). Pathological changes in the tissues were observed using optical microscopy. Lung injury, based on infiltration of inflammatory cells, pulmonary interstitial and alveolar edema, damage to alveolar structure and degree of fibrosis were assessed using the grading system reported by Szapiel *et al* (11). ALI was scored as follows (12): i) Alveolar congestion; ii) hemorrhage; iii) infiltration or aggregation of neutrophils in airspace or vessel wall, and iv) thickness of alveolar wall/hyaline membrane formation. Each item was scored on a five-point scale as follows: 0, minimal damage; 1, mild damage; 2, moderate damage; 3, severe damage; and 4, maximal damage. Repeatedly measured data were statistically analyzed using analysis of variance (ANOVA).

### Statistical analysis

Statistical analysis was performed with the SPSS version 15.0 (SPSS, Inc., Chicago, IL, USA). Data were analyzed for normality using the Kolmogorov-Smirnov method, and the normally distributed data were expressed as the mean ± standard deviation. To compare the normally distributed data between each group, one-way ANOVA followed by the Student-Newman-Keul’s post-hoc test was employed. P<0.05 was considered to indicate a statistically significant difference.

## Results

### M. suaveolens extract downregulates the expression of VEGF and NF-κB in lung tissue

**Figure 1:**
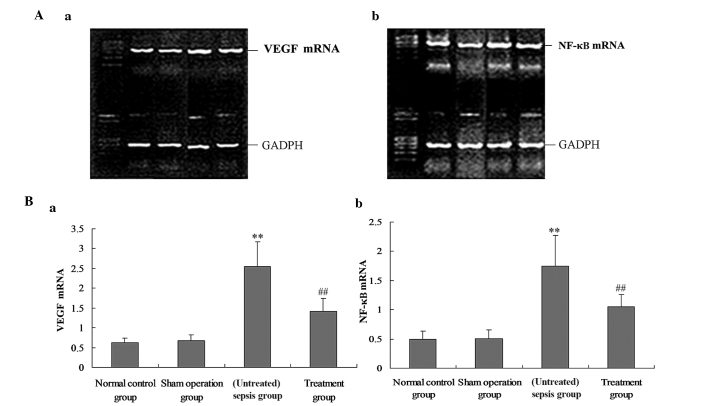
*M. suaveolens* extract blocked the expression of VEGF and NF-κB mRNA in lung tissue. Groups of mice were challenged with cecal ligation and puncture and treated with *M. suaveolens* extract 24 h later. The left lung tissues were homogenized and total RNA was extracted using TRIzol reagent and assayed by quantitative polymerase chain reaction. (A) Representative gels assessing (a) VEGF and (b) NF-κB levels are demonstrated. Lane 1, marker; lane 2, normal control group; lane 3, sham operation group; lane 4, (untreated) sepsis group and lane 5, treatment group. (B) Statistical summary of the densitometric analysis of VEGF and NF-κB mRNA expression in rats from the four groups; (a) VEGF mRNA and (b) NF-κB mRNA. Data are represented as the mean ± standard deviation of one experiment consisting of three replicates. ^**^P<0.01 vs. sham operation and normal control groups; ^##^P<0.01 vs. (untreated) sepsis group. VEGF, vascular endothelial growth factor; NF-κB, nuclear factor kappa B.

**Figure 2:**
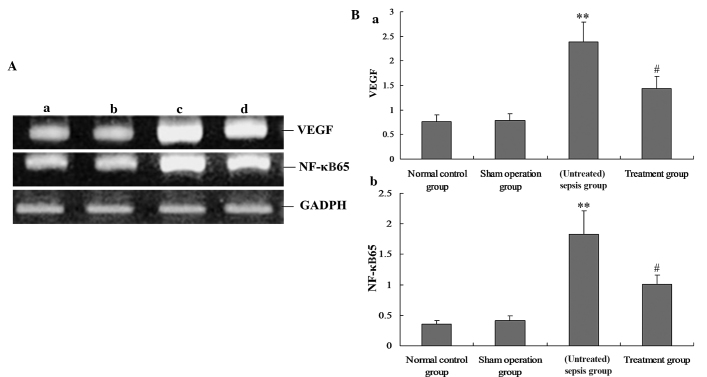
Effect of *M. suaveolens* extract on the expression of VEGF and NF-κB65 protein in lung tissue. Groups of mice were challenged with cecal ligation and puncture and treated with *M. suaveolens* extract 24 h later. The expression of VEGF, NF-κβ65 and GAPDH was detected by western blotting using specific antibodies. GAPDH protein was used an internal control. (A) Representative western blot analysis demonstrated the levels of VEGF and NF-κB65 protein expression in rats from the four groups; (a) normal control group; (b) sham operation group; (c) (untreated) sepsis group and (d) treatment group. (B) Quantification of the blots by densitometric analysis of (a) VEGF and (b) NF-κB65 protein expression in rats from the four groups. Data are presented as the mean ± standard deviation of one experiment consisting of three replicates. ^**^P<0.01 vs. the sham operation group and normal control group; ^#^P<0.05 vs*.* (untreated) sepsis group. VEGF, vascular endothelial growth factor; NF-κB, nuclear factor kappa B.

To investigate the underlying mechanism of the effect of *M. suaveolens* extract on CLP, the mRNA and protein expression of VEGF and NF-κB were measured by qPCR and western blot analysis, respectively. *In vivo*, the mRNA and protein expression levels of VEGF and NF-κB in the rat lung demonstrated significant increases during CLP-induced ALI (P<0.05; Figs. 1 and 2); however, these were significantly decreased in response to the administration of *M. suaveolens* extract (P<0.05; Figs. 1 and 2). A positive correlation was evident between the levels of VEGF and NF-κB mRNA (r=0.852, P<0.05) and between VEGF and NF-κB protein expression (r=0.794, P<0.01).

### Effect of M. suaveolens extract on lung localization of VEGF and NF-κB65 in CLP-induced ALI

**Figure 3:**
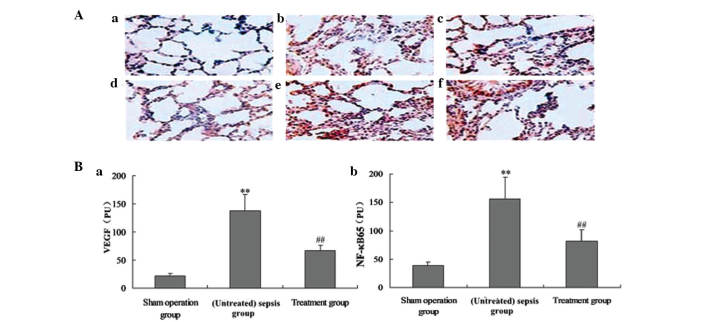
Effect of *M. suaveolens* extract on the protein expression of VEGF and NF-κB65 in rat lungs 24 h following cecal ligation and puncture-induced acute lung injury. Immunostaining was performed on lung sections following antigen retrieval using Retrievagen. (A) Representative immunostaining revealed VEGF and NF-κβ65-positive expression in rats from the four groups: (a–c) Expression of positive VEGF in the (a) sham operation group; (b) control group; (c) treatment group); (d–f) Expression of positive NF-κB65 in the (d) sham operation group; (e) (untreated) sepsis group; (f) treatment group (magnification, ×200). (B) Quantification of the images by densitometric analysis of (a) VEGF and (b) NF-κB-positive protein expression in rats from four groups. All values are expressed as the mean ± standard deviation. ^**^P<0.01 vs. the sham operation group; ^##^P<0.01 vs. (untreated) sepsis group. VEGF, vascular endothelial growth factor; NF-κB, nuclear factor kappa B.

Immunohistochemical analysis was used to determine the distribution of VEGF and NF-κB65 in rat lung 24 h following CLP or saline treatment. Positively immunostained cells appeared brown (Fig. 3A). VEGF and NF-κB65 were localized to the alveolar epithelium. The number of cells expressing VEGF and NF-κB65 was significantly increased in CLP-induced ACI, and this was significantly reduced by treatment with *M. suaveolens* extract (Fig. 3A and B).

### M. suaveolens extract decreases pro-inflammatory cytokines and VEGF production in CLP-induced rats

**Figure 4:**
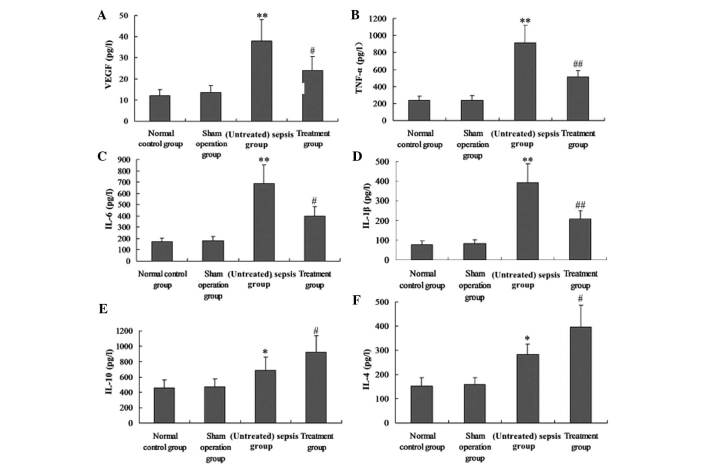
Effect of *M. suaveolens* extract on plasma levels of VEGF, TNF-α, IL-6, IL-1β, IL-4 and IL-10 levels in plasma. Groups of mice were challenged with cecal ligation and puncture, and treated with *M. suaveolens* extract 24 h later. (A) VEGF, (B) TNF-α, (C) IL-6, (D) IL-1β, (E) IL-10 and (F) IL-4 levels in plasma were determined by ELISA. Data are presented as the mean ± standard deviation of one experiment consisting of three replicates. ^*^P<0.05, ^**^P<0.01 vs. the sham operation group and normal control group; ^#^P<0.05, ^##^P<0.01 vs. (untreated) sepsis group. VEGF, vascular endothelial growth factor; NF-κB, nuclear factor kappa B; TNF-α, tumor necrosis factor-α; IL, interleukin.

**Figure 5:**
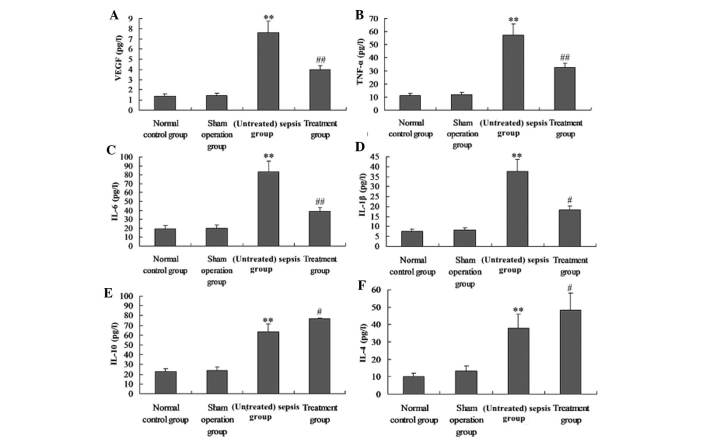
Administration of *M. suaveolens* extract attenuated lipopolysaccharide-induced pulmonary inflammation. Groups of mice were challenged with cecal ligation and puncture, and treated with *M. suaveolens* extract 24 h later. (A) VEGF, (B) TNF-α, (C) IL-6, (D) IL-1β, (E) IL-10 and (F) IL-4 levels in bronchoalveolar lavage were determined by ELISA. Data are presented as the mean ± standard deviation of one experiment consisting of three replicates. ^**^P<0.01 vs. the sham operation group and normal control group; ^#^P<0.05, ^##^P<0.01 vs. (untreated) sepsis group. VEGF, vascular endothelial growth factor; NF-κB, nuclear factor kappa B; TNF-α, tumor necrosis factor-α; IL, interleukin.

Serum and BAL were collected 24 h after the animals received CLP to evaluate the levels of VEGF, TNF-α, IL-1β, IL-6, IL-4 and IL-10. CLP caused a significant acute systemic inflammatory response and pulmonary capillary permeability, as demonstrated by the increased serum and BAL concentrations of the pro-inflammatory mediators TNF-α, IL-1β, IL-6 and VEGF. The presence of *M. suaveolens* extract reduced the increase of all of these pro-inflammatory mediators. CLP also caused an increase in the serum and BAL concentration of the anti-inflammatory cytokines IL-10 and IL-4. This change in IL-10 and IL-4 concentration was enhanced by the administration of *M. suaveolens* extract (Fig. 4E and F; Fig. 5E and F).

### Effect of M. suaveolens extract on pulmonary vascular permeability

**Figure 6:**
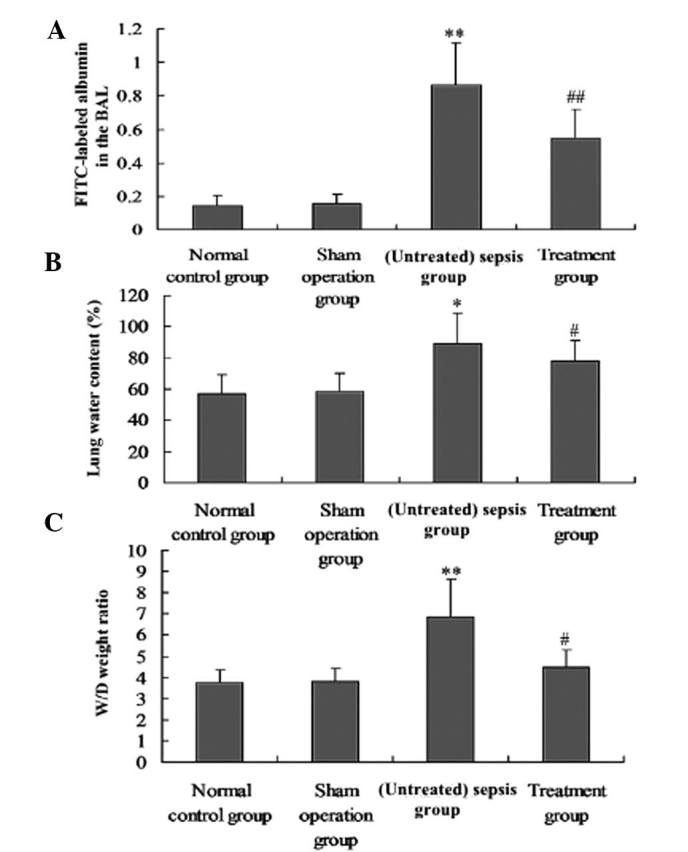
Administration of *M. suaveolens* extract reduces CLP-induced lung permeability. Rats were treated as indicated and (A) FITC-labeled albumin in the bronchoalveolar lavage fluid, (B) water content of lung tissue and (C) W/D lung weight ratio were determined 24 h following CLP challenge. Data are presented as the mean ± standard deviation of one experiment consisting of three replicates. ^*^P<0.05, ^**^P<0.01 vs. the sham operation and normal control groups; ^##^P<0.01 vs. (untreated) sepsis group. VEGF, vascular endothelial growth factor; NF-κB, nuclear factor kappa B; TNF-α, tumor necrosis factor-α; IL, interleukin; CLP, cecal ligation and puncture; W/D, wet/dry; FITC, fluorescein isothiocyanate.

*M. suaveolens* extract significantly reduced the CLP-induced increases in i.v. administered FITC-labeled albumin in BAL, as well as the W/D lung weight ratio and the water content of the lung tissue (P<0.05; Fig. 6A–C). The effects of CLP on pulmonary vascular permeability were also significantly suppressed by *M. suaveolens* extract (P<0.05; Fig. 6A–C).

### Effect of M. suaveolens extract on histological parameters of ACI

**Figure 7:**
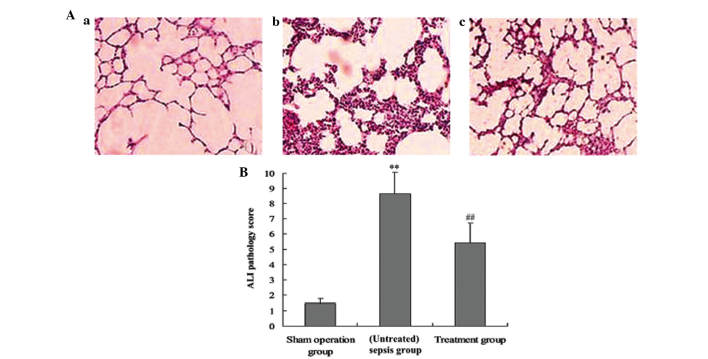
Administration of *M. suaveolens* extract ameliorated histopathologal changes in the lung tissue of CLP-ALI rats. The groups of rats were treated as described above and histological evaluation of the therapeutic potential of *M. suaveolens* extract on CLP-induced lung injury was performed 24 h following CLP challenge. (A) Representative images of hematoxylin and eosin-stained lung sections from three experimental groups (magnification, ×400): (a) Sham operation group; (b) (untreated) sepsis group; and (c) treatment group. (B) Lung injury score. ALI pathology score are expressed as the mean ± standard deviation of one experiment consisting of three replicates. ^**^P<0.01 vs. the sham operation group; ^##^P<0.01 vs. (untreated) sepsis group. CLP, cecal ligation and puncture; ALI, acute lung injury.

The lung tissue was significantly injured as indicated by the presence of intra-alveolar exudate, edema and inflammatory cell infiltration in the control group (Fig. 7A), and an increase in lung injury score (P<0.05, Fig. 7B). *M. suaveolens* extract significantly attenuated CLP-induced pathologic changes, demonstrating a significant decrease in lung injury score.

## Discussion

Numerous studies have demonstrated that the animal model of sepsis induced by the CLP method is highly stable and reproducible, and is applicable as a model of human sepsis (13). It is therefore currently regarded as the ‘gold standard’ for the study of sepsis. These animals demonstrate a hyperdynamic circulation and high metabolism in the early stage and a low dynamic circulation state at the later stage resembling the human condition; therefore, this is regarded as the sepsis model with the strongest clinical relevance (13). As a result, this CLP animal model of sepsis was adopted for the present study.

When sepsis occurs, a large number of inflammatory mediators are released(1,14–17); endothelial cells are impaired and contract, and the distance between endothelial cells is enlarged (14,15), all of which result in protein-rich fluid entering the mesenchymal cells from the blood vessels. This leads to a drop in the vascular endothelial colloidal osmotic pressure and an increase of colloidal osmotic pressure for tissue clearance, resulting in the presence of more water molecules among the organelles (16,17), and thus the development of interstitial edema. The diffusion distance for oxygen molecules from the blood capillary to tissue organs is increased, further aggregating hypoxia and leading to organ dysfunction (15,16). Furthermore, hypoxemia and hypoxia form a vicious cycle, resulting in further hypoxia-induced injury within the endothelial cells, leading to further capillary leakage (14). In the present study, CLP markedly enhanced the expression of pro-inflammatory mediators, including TNF-α and IL-6, and anti-inflammatory mediators, including IL-10, and increased lung capillary leakage. However, the administration of *M. suaveolens* extract significantly inhibited the expression of pro-inflammatory mediators, elevated the levels of anti-inflammatory mediators and reduced lung capillary leakage. Inhibition of inflammatory mediators by *M. suaveolens* therefore reduced the permeability of the lung capillary wall and reduced CLP-induced lung injury.

VEGF, a protein first obtained by isolation from *in vitro* cultures of bovine pituitary follicles in 1989, has specific mitogenic effects on vascular endothelial cells which promote endothelial cell proliferation, increase microvascular permeability and promote the growth of endothelial cells within the blood and lymphatic vessels (18). VEGF is therefore a survival factor existing in endothelial cells. VEGF has been used in numerous fields since its discovery, including as a marker for clinical tumor prognosis, early diagnosis of acute myocardial ischemia and evaluation of bronchial asthma (19). In recent years, its role in sepsis blood capillary leakage has drawn increasing attention (5,19,20). VEGF has a significant role among the known microvascular permeability inducers, and effects ~50,000-fold of those of histamine have been observed, with activities at concentrations <1 nmol/l. Such effects are not inhibited by antihistamines, platelet-activating factor inhibitors or other inhibitors of inflammation (5). An excessive increase of vascular permeability may cause blood flow into the tissue, leading to poor blood supply for visceral function, eventually resulting in organ dysfunction. van der Flier et al (19) suggested that an increase in capillary permeability is a key factor in the occurrence and development of sepsis, while VEGF is the key molecule for controlling vascular permeability, and is therefore a potential factor that leads to inflammation-associated capillary permeability. For this reason, VEGF was used as a measurement index in the present study. The results indicated that increased expression of NF-κB promotes the expression and production of VEGF, and significantly aggravates pathological lung damage in rats with sepsis.

It has been demonstrated that through its involvement in the transcription of a variety of cytokine genes, NF-κB has a complex and important role in regulating the inflammatory network (21). Activated NF-κB may increase the transcription of numerous cytokines, including TNF-α and IL-1, thus rapidly increasing the quantity of inflammatory factors synthetized by inflammatory cells (22,23). Therefore, blocking the activation of NF-κB may facilitate controlling the blood capillary permeability of lung tissue, and thus attenuate lung microvascular leakage and ALI in rats with sepsis. It is observed from this study that inhibition of NF-κB activity by *M. suaveolens* significantly reduced lung inflammatory responses, decreased the expression of VEGF and alleviated capillary permeability in rats with sepsis.

Vascular leakage in multiple organs is a characteristic pathological change in sepsis (24). In the present study, the W/D ratio of the lung was firstly examined. It was identified that salidroside treatment attenuates the development of pulmonary edema, as demonstrated by a significant decrease in lung W/D ratio. Another index of ALI by CLP is the total protein concentration in the BAL fluid which indicated epithelial permeability and pulmonary edema. FITC-labeled albumin, a macromolecular marker, is widely used to evaluate pulmonary microvascular permeability (25), and FITC-labeled albumin was therefore injected and assessed in the BAL in the present study. As expected, CLP was identified to cause a significant increase in BAL fluid protein concentration, lung W/D ratio and FITC-labeled albumin. CLP-induced increases in total protein in the BAL fluid, lung W/D ratio and FITC-labeled albumin were inhibited by *M. suaveolens*. In the present study, it was also identified that *M. suaveolens* significantly reduced the accumulation and sequestration of activated macrophages, suppressed formation of pulmonary edema, prevented an increase in septa thickness and ameliorated the lung capillary leakage in rats with sepsis.

Capillary leakage and endothelial dysfunction are increasingly recognized to significantly contribute to organ failure and death in sepsis and systemic inflammation (26–28). Therapeutic targeting of capillary leakage in sepsis and systemic inflammation is therefore considered a highly relevant clinical approach. The pharmacological effects of *M. suaveolens* (8,29–34) indicate that one of the active components, coumarin, is able to reduce capillary permeability and vascular resistance (8,29), increase vein tension and improve circulation (30,31), so that the open arteriovenous anastomosis tubes are closed to reduce local congestion, therefore reducing soft tissue bleeding. Coumarin may also inhibit the release of a variety of vascular active substances (32), suppressing adenosine diphosphate and collagen-induced platelet aggregation as well as the release of 5-hydroxytryptamine, platelet factor 4, thromboxane A2 and platelet-derived growth factor from the platelet (33,34). These effects prevent the loss of serum, maintain normal colloid osmotic pressure and have anti-edema effects, therefore reducing post-traumatic swelling. The tannic acid component of *M. suaveolens* inhibits the synthesis of prostaglandins and other inflammatory mediators, as well as reducing pain and the inflammatory reaction, reducing vascular permeability and leakage from tissue clearance. Other effects include increasing the formation of newborn granulation tissue, promoting wound healing (closing the wound and preventing secondary wound exudate), expanding lymphatic vessels, increasing lymph flow, accelerating lymph circulation and reducing soft tissue swelling. Medicinal chemistry study results have identified that *M. suaveolens* contains coumarin, flavonoids, phenolic acids, saponins and other substances that have effective anti-inflammatory and antibacterial activities (31). Although its effect of reducing the blood capillary leakage has been reported, studies of how this occurs at the molecular level remain limited. The present study revealed that *M. suaveolens* is able to inhibit the activation and expression of NF-κB in rats with sepsis, reduce the generation of inflammatory mediators, including TNF-α and IL-6 (with consequent reduction of VEGF expression), therefore decreasing lung capillary permeability and having a protective role in ALI.

Although an ability of *M. suaveolens* to reduce blood capillary leakage has been reported in clinical practice, the molecular biological mechanisms mediating this effect have not been well investigated. The results of the present study suggest a novel compensatory mechanism for the action of *M. suaveolens* in the maintenance of lung vascular permeability under pathological inflammatory conditions, through downregulating VEGF expression. To the best of our knowledge, the present study demonstrated for the first time the role of *M. suaveolens* in mediating anti-inflammatory and barrier protective effects of lung capillary permeability in a model of ALI. Therefore, the beneficial effects of *in vivo* administration of *M. suaveolens* on the parameters of capillary permeability in lung injury described in this study may represent a novel therapeutic modality for the treatment of CLP-induced lung capillary permeability and ALI.

## Abbreviations

VEGF: vascular endothelial growth factor
NF-κB: nuclear factor kappa B
IL-6: interleukin-6
TNF-α: tumor necrosis factor-α
LPS: lipopolysaccharide
PCR: polymerase chain reaction
SIRS: systemic inflammatory response syndrome
CLP: cecal ligation and puncture
